# The Association of Human Apolipoprotein C-III Sialylation Proteoforms with Plasma Triglycerides

**DOI:** 10.1371/journal.pone.0144138

**Published:** 2015-12-03

**Authors:** Hussein N. Yassine, Olgica Trenchevska, Ambika Ramrakhiani, Aarushi Parekh, Juraj Koska, Ryan W. Walker, Dean Billheimer, Peter D. Reaven, Frances T. Yen, Randall W. Nelson, Michael I. Goran, Dobrin Nedelkov

**Affiliations:** 1 Department of Medicine, University of Southern California, Los Angeles, United States of America; 2 The Biodesign Institute, Arizona State University, Tempe, AZ, United States of America; 3 Department of Medicine, Phoenix Veteran Affairs Medical Center, Phoenix, AZ, United States of America; 4 Department of Preventive Medicine, University of Southern California, Los Angeles, United States of America; 5 Department of Preventive Medicine, Icahn School of Medicine at Mount Sinai, New York, NY, United States of America; 6 Biostatics Consulting Lab, University of Arizona, Tucson, AZ, United States of America; 7 Université de Lorraine, URAFPA, INSERM, Vandoeuvre-lès-Nancy, France; Katholieke Universiteit Leuven, BELGIUM

## Abstract

**Introduction:**

Apolipoprotein C-III (apoC-III) regulates triglyceride (TG) metabolism. In plasma, apoC-III exists in non-sialylated (apoC-III_0a_ without glycosylation and apoC-III_0b_ with glycosylation), monosialylated (apoC-III_1_) or disialylated (apoC-III_2_) proteoforms. Our aim was to clarify the relationship between apoC-III sialylation proteoforms with fasting plasma TG concentrations.

**Methods:**

In 204 non-diabetic adolescent participants, the relative abundance of apoC-III plasma proteoforms was measured using mass spectrometric immunoassay.

**Results:**

Compared with the healthy weight subgroup (n = 16), the ratios of apoC-III_0a_, apoC-III_0b_, and apoC-III_1_ to apoC-III_2_ were significantly greater in overweight (n = 33) and obese participants (n = 155). These ratios were positively correlated with BMI z-scores and negatively correlated with measures of insulin sensitivity (S_i_). The relationship of apoC-III_1_ / apoC-III_2_ with S_i_ persisted after adjusting for BMI (p = 0.02). Fasting TG was correlated with the ratio of apoC-III_0a_ / apoC-III_2_ (r = 0.47, p<0.001), apoC-III_0b_ / apoC-III_2_ (r = 0.41, p<0.001), apoC-III_1_ / apoC-III_2_ (r = 0.43, p<0.001). By examining apoC-III concentrations, the association of apoC-III proteoforms with TG was driven by apoC-III_0a_ (r = 0.57, p<0.001), apoC-III_0b_ (r = 0.56. p<0.001) and apoC-III_1_ (r = 0.67, p<0.001), but not apoC-III_2_ (r = 0.006, p = 0.9) concentrations, indicating that apoC-III relationship with plasma TG differed in apoC-III_2_ compared with the other proteoforms.

**Conclusion:**

We conclude that apoC-III_0a_, apoC-III_0b_, and apoC-III_1_, but not apoC- III_2_ appear to be under metabolic control and associate with fasting plasma TG. Measurement of apoC-III proteoforms can offer insights into the biology of TG metabolism in obesity.

## Introduction

Apolipoprotein C-III (apoC-III) is a protein of 79 amino acids that is synthesized in the liver and, to a lesser degree in the intestine, and regulates triglyceride (TG) metabolism [1]. It is primarily located on the surface of lipoproteins [2]. In the circulation, apoC-III is a constituent of both apoB and apoA-I containing lipoproteins. The majority of apoC-III is found on the HDL fraction in normolipidemic individuals and on triglyceride-rich lipoproteins in patients with elevated levels of plasma triglyceride [3]. ApoC-III plays a pivotal role in regulating the plasma metabolism of VLDL, IDL, and LDL, primarily by inhibiting receptor-mediated uptake of these lipoproteins by the liver [4]. Overexpression of apoC-III in transgenic mice leads to severely increased plasma TG levels [5]. Mutations that disrupt apoC-III expression and function in humans are associated with lower plasma TG and apoC-III levels, and lower risk of coronary artery disease [6].

Overproduction of apoC-III and of apoB lipoproteins that contain apoC-III is a common feature of patients with obesity and hypertriglyceridemia [7, 8]. Insulin and glucose regulate apoC-III expression [9–11]. Treatment of insulin-deficient diabetic mice with insulin resulted in a 2.5-fold decrease in hepatic apoC-III mRNA levels and a corresponding decrease in apoC-III gene transcriptional activity [11]. Insulin treatment of HepG2 cells transfected with an apoC-III luciferase reporter construction caused a dose-dependent two-fold reduction in apoC-III transcriptional activity [11]. A genetic variant form of the human apoC-III promoter, containing five single base pair changes that makes it less responsive to insulin, has been shown to be associated with severe hypertriglyceridemia [10]. Glucose can also induce apoC-III transcription in primary rat hepatocytes and immortalized human hepatocytes via a mechanism involving the transcription factors carbohydrate response element–binding protein and hepatocyte nuclear factor-4 [9]. Lowering of apoC-III by antisense oligonucleotides, reduces fasting and post prandial triglyceride levels [12, 13]. Thus, apoC-III is induced in obesity by dysregulation of insulin and glucose signaling, and is intricately involved in establishing hypertriglyceridemia.

ApoC-III in plasma exists in multiple proteoforms. The most common proteoforms differ by their sialic acid content: apoC-III_0_, apoC-III_1_ and apoC-III_2_ containing 0, 1, and 2 molecules of sialic acid per molecule of protein, respectively [14–16]. In plasma from healthy volunteers, apoC-III_0_, apoC-III_1_ and apoC-III_2_ comprise approximately 22, 45, and 33% of the total apoC-III, respectively [17, 18]. Sialylation of apoC-III occurs in the Golgi compartment by the activity of sialyltransferases [19], whereas de-sialylation of this protein is mediated by lysosomal neuraminidase [20]. ApoC-III sialylation appears to be under metabolic control. For example, reduced apoC-III_1_ to apoC-III_2_ ratio was observed following weight loss by caloric restriction [21] or bariatric surgery [22]. In contrast, increased apoC-III_0_ was demonstrated after carbohydrate feeding [23, 24], in familial combined hyperlipidemia [25], and in metabolic syndrome [26].

Progress toward understanding the importance of apoC-III sialylations in TG metabolism *in vivo* has been hindered by the lack of a robust method to measure these sialylated proteoforms in plasma. Traditionally, sialylation of apoC-III has been studied using isoelectric focusing, a time sensitive method that is not amenable for use in large studies. In addition, isoelectric focusing can only resolve three or four apoC-III proteoforms based on mass and charge [24, 27–29]. Mass spectrometric immunoassay (MSIA) is a high throughput methodology that is utilized to identify and quantify molecular variants and posttranslational modifications of plasma proteins. MSIA is based on the isolation of protein moieties from a biological milieu by immobilized antibodies, which is followed by mass spectrometry detection. In our previous work using MSIA, we identified numerous proteoforms originating from apolipoprotein A-I, A-II, C and serum amyloid A [30–33]. In this study, our objectives were to isolate and identify the different apoC-III sialylated forms by MSIA, and determine the relationship between these proteoforms with fasting plasma TG levels.

## Materials and Methods

### Reagents

Affinity purified polyclonal goat anti-human antibodies to apoC-I (Cat. No. 31A-G1b), apoC-II (32AG2b), apoC-III (33A-G2b), and HRP Goat Anti-Human apoC-III (33H-G2a), apoC-II (32H-G4a) were obtained from Academy Bio-medical Co. (Houston, TX, USA). Protein calibration standard I (Cat. No. 206355) was purchased from Bruker (Billerica, MA). Phosphate buffered saline (PBS) buffer (Cat. No. 28372), MES buffered saline (28390), acetonitrile solution (ACN; A955-4), hydrochloric acid (HCl; A144-212), *N*-methylpyrrolidinone (NMP; BP1172-4), 1,1’ Carbonyldiimidazole (97%) (CDI, 530-62-1), affinity pipettes fitted with porous micro columns (991CUS01) were obtained from Thermo Scientific (Waltham, MA, USA). Tween20 (Cat. No. P7949), trifluoracetic acid (TFA, 299537), sinapic acid (85429-5G), sodium chloride (S7653), HEPES (H3375), ethanolamine (ETA; 398136) were obtained from Sigma Aldrich (St. Louis, MO, USA). Acetone (Cat. No. 0000017150) was obtained from Avantor Performance Materials (Center Valley, PA, USA).

### Mass spectrometric immunoassay

Analysis of apoC-III was performed using triplexed mass spectrometric immunoassay (MSIA) for analysis of apoC-I, apoC-II and apoC-III, as previously described [33]. In short, affinity pipettes were derivatized with corresponding antibodies (0.4, 2.25 and 2.5 μg of anti-apoC-I, anti-apoC-II and anti-apoC-III and 0.8 μg anti-lysozyme per pipette respectively). Following sample preparation (total of 120 μL of plasma sample, diluted 120-fold in PBS,0.1%Tween), Multimek 96-channel robot was used to capture apoC-III proteoforms from each analytical sample by repeated aspirations and dispensing cycles through the pipettes. After rinsing the non-specific bounded proteins, captured apolipoproteins were eluted directly onto a 96-well formatted MALDI target using a sinapinic acid matrix. Linear mass spectra were acquired from each sample spot using an Ultraflex III MALDI-TOF instrument (Bruker, Billerica, MA) operating in positive ion mode. An average of 5000 laser shots mass spectra was saved for each sample spot. Mass spectra were internally calibrated using protein calibration standard-I, and further processed with Flex Analysis 3.0 software (Bruker Daltonics). All peaks representing apolipoproteins and their proteoforms as well as the signals from lysozyme (used as internal reference standard and spiked in constant concentration in all samples) were integrated baseline-to-baseline using Zebra 1.0 software (Intrinsic Bioprobes Inc). All peaks were normalized towards the lysozyme signal. Peaks that were not resolved at baseline were integrated manually. In addition, the peak areas were corrected individually with baseline noise-bin signals. To assess for the consistency of the ionization efficiency and reproducibility between and within runs, a control plasma sample was run in triplicate with each analysis. Although ssamples for these analyses were stored for up to 12 years at -80°C before measurements of apoC proteoforms were performed, previous investigation of the effects of storage, time and freeze/thaw cycles on these assays indicated that the measurements are relatively stable [33].

### ELISA

Sandwich ELISA using apoC-III antibodies and apoC-III protein standard obtained from Academy Biomedical was performed as previously described [8]. The inter- and intra-assay coefficients of variation were less than 10%.

### Clinical Samples

Adolescent Hispanic children (8–17 years of age) without type two diabetes (n = 204) were recruited. Samples were obtained from a variety of studies using essentially identical protocols and measures conducted by the University of Southern California Childhood Obesity Research Center over the past 15 years. BMI percentiles and z-scores were calculated as described [34]. The study group was divided by BMI percentiles into three groups: healthy weight (<85^th^ percentile, n = 16), overweight (between 85^th^ and 95^th^ percentile, n = 33) and obese (>95^th^ percentile, n = 155). Blood was collected for clinical laboratory measurements (lipid profile, liver function tests, and insulin-modified frequently sampled intravenous glucose tolerance test for measuring insulin sensitivity-S_i_ [35] in a subset of these participants, Table 1) after an overnight fast (10 hours). Additional samples were collected in EDTA tubes, and plasma from these samples was separated and immediately frozen at -80°C for all other assays. Demographic information (sex, age, and ethnicity), physical exam measurements (height, weight, and body mass index-BMI), medication use, and medical history (hypertension, hyperlipidemia, and smoking) were recorded. Exclusion criteria included the following: (i) met any diagnostic criteria for diabetes; (ii) the use of medications or supplements or the past or present diagnosis of other syndromes or diseases known to influence liver function, insulin action, or lipid levels; (iii) previous diagnosis of any major illness since birth; (iv) smoking (currently smoked, or had smoked more than 100 cigarettes in their lifetime) or drinking alcohol on a regular basis (in excess of two drinks per week as determined by a questionnaire; (v) or involvement in any weight loss/exercise/sports program currently or within 6 months prior to participation.

**Table 1:** The clinical and biochemical characteristics of the study population by sex.

	Males (n = 84)	Females (n = 120)
Age (years)	14 (2)	13 (3)
BMI (kg/m^2^)	32 (7)	30 (8)
BMI z -score	2.1 (1.75, 2.42)	2.0 (1.50, 2.33)
Fasting glucose (mg/dL)*	91 (7)	92 (13)
Fasting Insulin (IU) * †	15 (13)	22 (12)
Insulin Sensitivity (S_i_) [X10^4^ min^-1^/(pmol/l)]*	1.56 (0.94, 2.60)	1.58 (1.1, 2.3)
TG (mg/dL) *	101 (75, 136)	91 (64, 125)
Total Cholesterol (mg/dL)*	140 (27)	139 (29)
HDL Cholesterol (mg/dL)*	36 (7)	38 (10)
LDL Cholesterol (mg/dL)*	81 (26)	88 (30)

Data are presented as means (SD) or median (25^th^, 75^th^ percentile) for non-normally distributed data. The differences between males and females in age, BMI or lipid measures were not significant.

*Fasting glucose, n = 120, Fasting insulin, n = 119, Si, n = 117, TG, n = 173, cholesterol, n = 132.

^†^ p = 0.005. IU: International Units. TG: Triglycerides.

### Study approval

This study was approved by the University of Southern California (USC) Institutional Review Board. Written informed consent was obtained from parents or legal guardians of minors. Additionally, child assent was obtained in writing.

### Statistical Analysis

Mean (SD) or median (25^th^ and 75^th^ percentiles) for non-normally distributed data were calculated for continuous variables. The study subgroups were compared using ANOVA. The sialylation ratios between the groups were modeled using logistic regression adjusting for age and sex. The relation between apoC-III proteoform ratios with BMI z-scores, S_i_, or TG concentrations was analyzed using Spearman correlation coefficient. To test if apoC-III concentrations and apoC-III_1_/apoC-III_2_ ratio were independently associated with TG concentrations, a linear regression model was used, and apoC-III concentrations and ratio were centered to facilitate interpretation of the results. Centering the regressors allowed the coefficients to represent the change in TG associated with one SD change in the predictor. Statistical analyses used R program version 3.3. p<0.05 was defined as significant.

## Results

The study participants were predominantly overweight and obese Hispanic adolescents with a BMI z-score between– 0.98 and 3.1, and fasting plasma TG levels ranging from 19 to 330 mg/dL. None of the recruited participants had been diagnosed with diabetes, nor were on diabetes or lipid-lowering therapies. The mean fasting glucose among the participants was 92 mg/dL. With the exception of fasting insulin, the characteristics of the study participants did not differ by sex. Additional clinical and biochemical characteristics are summarized in Table 1.

**Fig 1:**
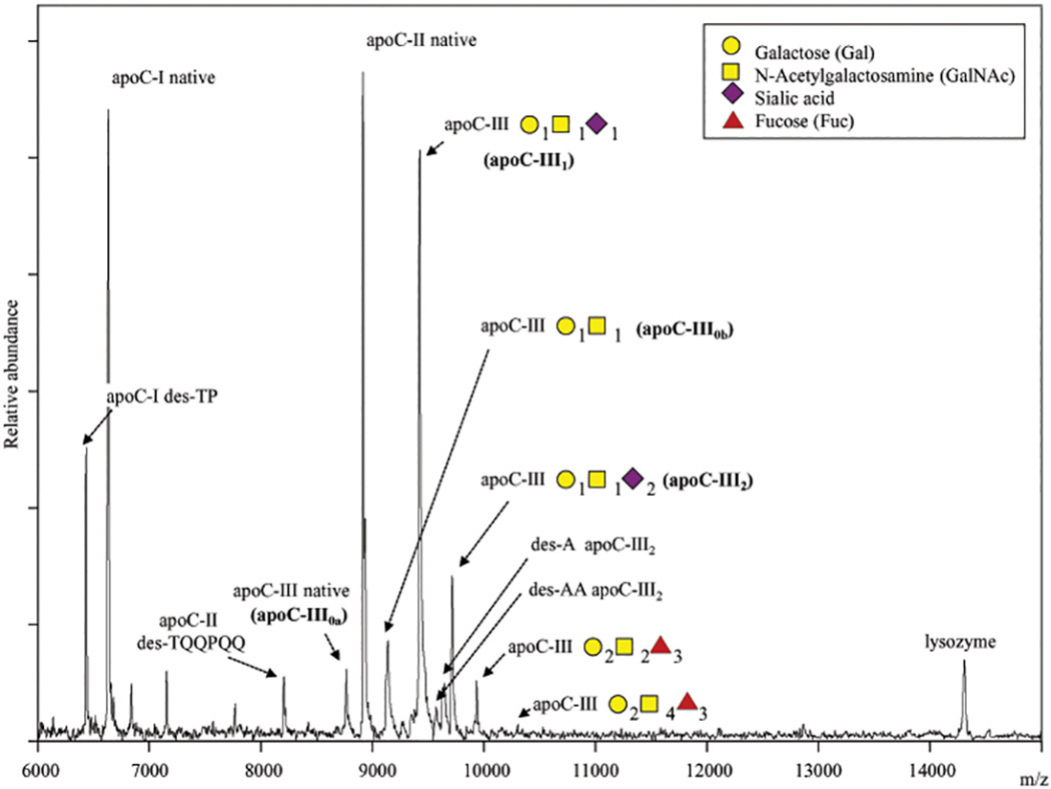
Mass spectra of plasma apolipoproteins C-I, C-II and C-III and their proteoforms resolved using MSIA loaded with antibodies for apoC-I, C-II and C-III. Two proteoforms of apoC-I (native and TP truncated), and two proteoforms of apoC-II (native and TQQPQQ truncation) can be detected. 12 different proteoforms of apoC-III can be resolved. This study was focused on four proteoforms of apoC-III: native apoC-III (apoC-III_0a_), glycosylated apoC-III (apoC-III_0b_), monosialylated (apoC-III_1_), and disialylated (apoC-III_2_).

The relative abundance of apoC-I, C-II and C-III proteoforms in fasting plasma was assessed by MSIA (Fig 1). A total of 12 apoC-III proteoforms were detected, reflecting variations in galactose (Gal), N-acteyl galactosamine (GalNAc), fucose, alanine truncations or sialic acid residues. Because of the higher abundance and potential functional importance of the sialylations, analysis of apoC-III was largely restricted to these proteoforms. In addition, MSIA was utilized to measure apoC-I and C-II proteins. Characteristics of apoC-I, C-II and C-III proteoforms by MSIA are summarized in Table 2. In contrast to isoelectric focusing, MSIA can resolve two asialylated proteoforms based on mass (apoC-III_0a_, and apoC-III_0b_) with apoC-III_0b_ having a galactose and GalNAc residues. ApoC-III_0a_ is referred here as the native apoC-III, and corresponds to the full-length sequence of unmodified apoC-III protein (MW = 8765 Da). As shown in Fig 1, the most abundant proteoform of apoC-III is the mono-sialylated (apoC-III_1_: 52.5± 3.6 peak area ratio to total apoC-III peak area), followed by the di-sialylated protein (apoC-III_2_: 12.4 ± 3.8 peak area ratio), with the native proteoform being less in abundance (apoC-III_0a_: 6.9 ± 3.5 peak area ratio). In this study, apoC-III_0b_ was relatively greater in abundance than apoC-III_0a_ (apoC-III_0b_: 22.3 ± 3.3 peak area ratio).

**Table 2:** ApoC-I, apoC-II and apoC-III proteoforms detected by MSIA.

Theoretical m/z value	Observed m/z value	Proteoform	Label
6432.3	6432.1	ApoC-I lacking *N*-terminal dipeptide (des-TP)	ApoC-I des-TP
6630.6	6630.6	Full length apoC-I form	ApoC-I native
8204.2	8204.8	ApoC-II lacking *N*-terminal hexapeptide (des-TQQPQQ)–termed mature apoC-II	ApoC-II des-TQQPQQ
8764.7	8765.3	Full length apoC-III form	ApoC-III_0a_ (native)
8914.9	8914.7	Full length apoC-II form—termed pro-apoC-II	ApoC-II native
9135.8	9136.2	Asialylated apoC-III—apoC-III+ (Gal)_1_(GalNAc)_1_	ApoC-III_0b_
9422.2	9422.9	Mono-sialylated apoC-III—apoC-III + (Gal)_1_(GalNAc)_1_(NeuAc)_1_	ApoC-III_1_
9712.5	9712.6	Di-sialylated apoC-III—apoC-III + (Gal)_1_(GalNAc)_1_(NeuAc)_2_	ApoC-III_2_

Gal—galactose; GalNAc—N-acetylgalactosamine; NeuAc—N-acetyl neuraminic acid (sialic acid)

**Fig 2:**
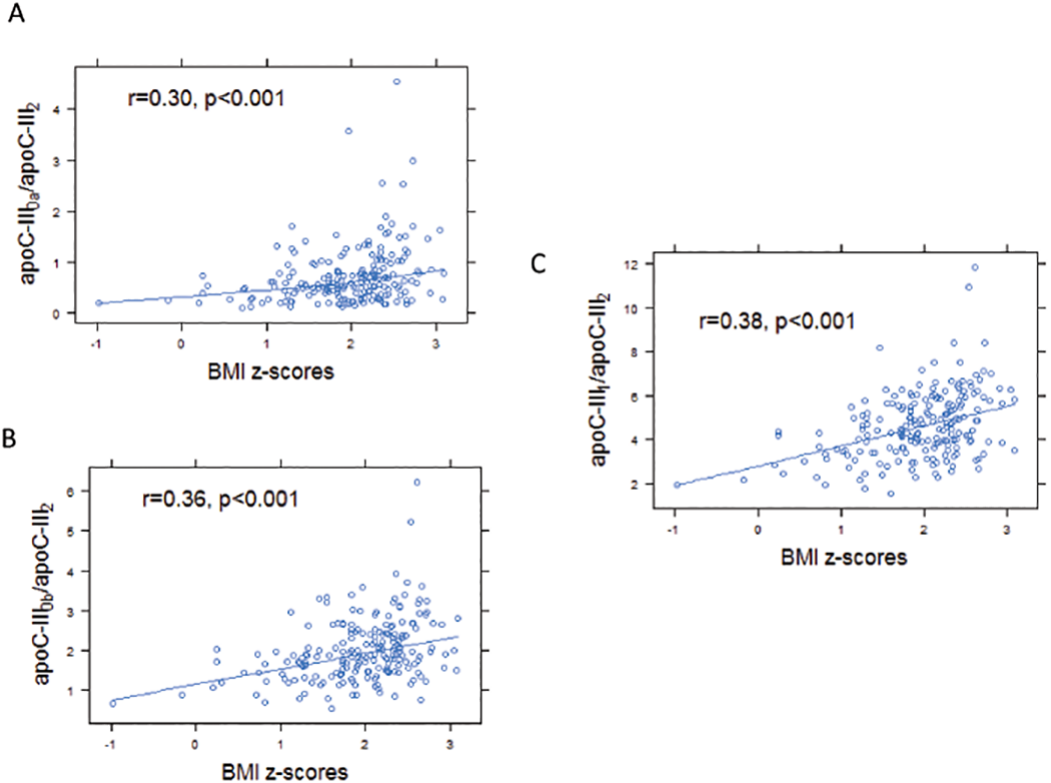
BMI z-scores were associated with greater ratio of apoC-III_0_ and apoC-III_1_ proteoforms to apoC-III_2_ in plasma (n = 204). BMI z-scores correlated with apoC-III_0a_ / apoC-III_2_ ratio (r = 0.30, p<0.001, A), apoC-III_0b_ / apoC-III_2_ ratio (r = 0.36, p<0.001, B), and apoC-III_1_ / apoC-III_2_ ratio (r = 0.38, p<0.001, C).

**Fig 3:**
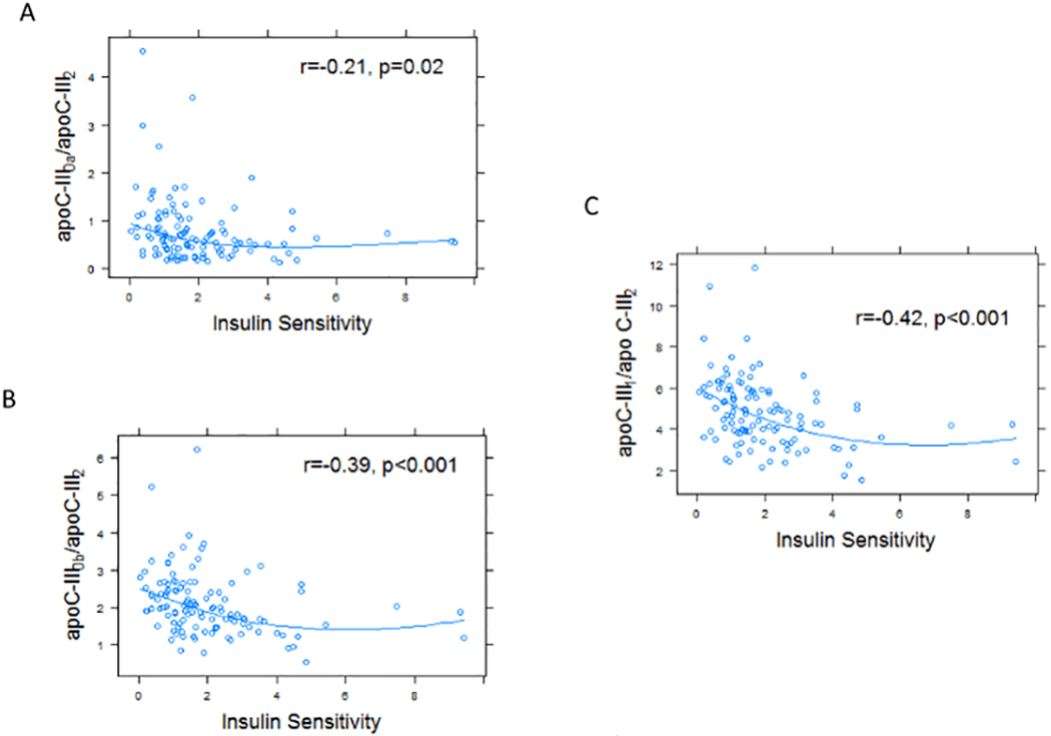
Insulin sensitivity measures (S_i_) were associated with lower ratios of apoC-III_0_ and apoC-III_1_ proteoforms to apoC-III_2_ in plasma. S_i_ was measured in a subset of participants (n = 117). S_i_ negatively correlated with apoC-III_0a_ / apoC-III_2_ ratio (r = -0.21, p = 0.02, A), apoC-III_0b_ / apoC-III_2_ ratio (r = -0.39, p<0.001, B), and apoC-III_1_ / apoC-III_2_ ratio (r = -0.42, p<0.001, C).

The study group was divided into 3 categories based on BMI percentiles. The three categories included participants with a healthy weight (n = 16), overweight (n = 33) and obese (n = 155) adolescents. The participants in the healthy weight group were younger in age with fewer males compared to the two other subgroups. Obesity was associated with decreases in both insulin sensitivity (S_i_) and HDL cholesterol concentrations, and increases in TG concentrations. These data are summarized in Table 3. The ratios apoC-III_0a_, apoC-III_0b_ and apoC-III_1_ to apoC-III_2_ were significantly greater in the overweight and obese groups compared to the healthy weight group (Table 3). These ratios were positively correlated with BMI z-scores (Fig 2A, 2B and 2C), and negatively correlated with measures of insulin sensitivity (Fig 3A, 3B and 3C). The relationship between apoC-III_1_ / apoC-III_2_ ratio and insulin sensitivity persisted after adjusting for BMI, age and sex (R^2^ = 0.19, p = 0.02). Fasting glucose levels did not significantly correlate with the apoC-III proteoform ratios (p>0.1).

**Table 3:** The clinical and biochemical characteristics of the study population by weight groups.

	Healthy Weight (<85^th^ percentile, n = 16)	Overweight (85-95^th^ percentile, n = 33)	Obese (> 95^th^ percentile, n = 155)	p value (group)
Age*	10 (2)	14 (3)	14 (3)	<0.001
Male/Female (n)**	2/14	12/21	70/85	0.06
Weight (kg)***	36 (10)	62 (23)	87 (24)	<0.001
BMI (z-scores)***	0.73 (0.30,0.86)	1.32 (1.25, 1.5)	2.25 (1.92,2.43)	<0.001
Fasting glucose (mg/dL)	83 (12)	91 (6)	92 (10)	0.19
Fasting Insulin (IU) ****	5 (5)	9 (5)	19 (13)	0.001
Insulin Sensitivity (S_i_) [X10^4^ min^-1^/(pmol/l)]*	6.0 (4.1, 8.0)	2.5 (2.1, 3.6)	1.4 (0.87, 2.2)	<0.001
TG (mg/dL)^†^	58 (50,74)	78 (64, 98)	111 (76, 146)	<0.001
Total Cholesterol (mg/dL)	118 (17)	128 (30)	143 (27)	0.03
HDL Cholesterol (mg/dL)*	48 (13)	39 (8)	36 (8)	<0.001
LDL Cholesterol (mg/dL)	86 (29)	84 (34)	85 (27)	0.98
ApoC-III_0a_/ apoC-III_2_ *	0.29 (0.24,0.50)	0.51 (0.39, 0.95)	0.63 (0.40, 0.96)	<0.001
ApoC-III_0b_/ apoC-III_2_ ^†^	1.41 (1.16, 1.72)	1.71 (1.24, 2.01)	1.97 (1.56, 2.53)	<0.001
ApoC-III_1_/ apoC-III_2_ ^†^	3.32 (2.7, 3.6)	4.23 (3.0, 5.1)	4.82 (3.9, 5.8)	<0.001

BMI percentile categories were based on BMI z-scores. Data are presented as means (SD) or median (25^th^, 75^th^ percentile) for non-normally distributed data. The data was analyzed by ANOVA followed by groupwise comparisons. Significance was defined with a p<0.05. The differences of the apoC-III ratios among the three groups were adjusted for age and sex using logistic regression.

* healthy weight group was significantly different from the overweight and obese groups

** less males than females in healthy weight compared to the overweight and obese groups

*** three groups were significantly different

**** obese group was significantly different from the overweight group

^†^ obese group was significantly different from overweight and healthy groups

**Fig 4:**
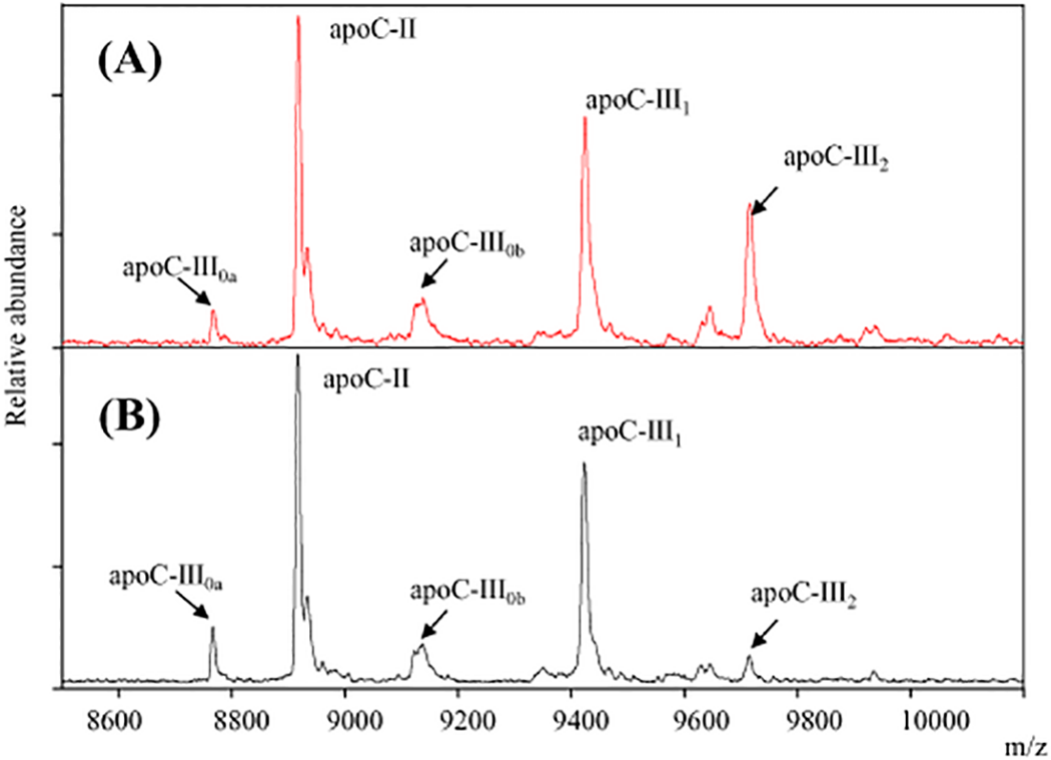
Representative MSIA mass spectra from two participants of the upper and lower quartile of TG concentrations. Participant 1 (upper panel) is a 15 years old male with a BMI z-score of 1.6, TG of 36 and HDL of 41 mg/dL. In contrast, participant 2 (lower panel) is a 14 years old male with a BMI z-score of 2.73, TG of 224 and HDL of 36 mg/dL. Compared to the participant with lower TG concentrations, mass spectrum from the individual with hypertriglyceridemia reveals a greater ratio of apoC-III_0a_, apoC-III_0b_, and apoC-III_1_ to apoC-III_2_.

**Fig 5:**
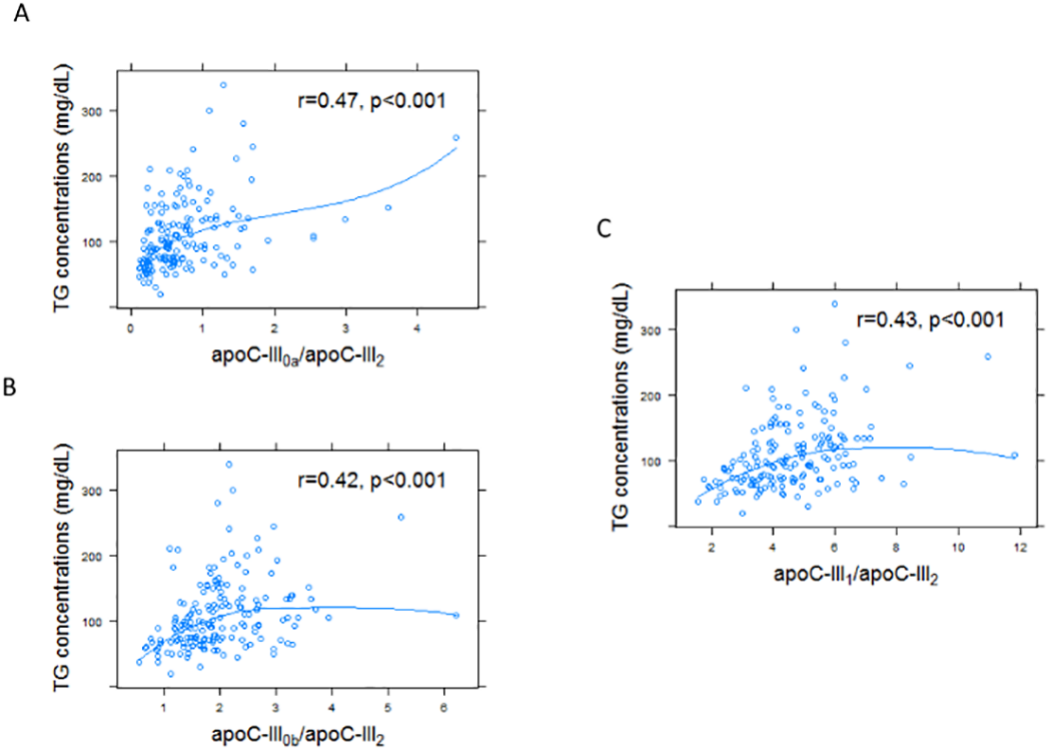
The ratio of apoC-III_0_ and apoC-III_1_ proteoforms to apoC-III_2_ in plasma was positively correlated with fasting plasma TG concentrations (n = 173). TG concentrations correlated with apoC-III_0a_ / apoC-III_2_ ratio in plasma (r = 0.47, p<0.001, A), and apoC-III_0b_ / apoC-III_2_ ratio (r = 0.42, p<0.001, B) and C-III_1_ / apoC-III_2_ ratio (r = 0.43, p<0.001, C) in plasma.

ApoC-III_2_ had a different relationship with fasting TG than the other apoC-III proteoforms. Representative MSIA mass spectra from two participants in the upper and lower quartiles of TG levels are presented in Fig 4. Compared to the normotriglyceridemic participant, mass spectrum from the hypertriglyceridemic individual revealed a greater ratio of apoC-III_0a_, apoC-III_0b_, or apoC-III_1_ to apoC-III_2._ In all the participants, the ratios of apoC-III_0a_ / apoC-III_2_, apoC-III_0b_ / apoCIII_2_ and apoC-III_1_ / apoC-III_2_ were significantly correlated with greater fasting TG (Fig 5A, 5B and 5C). Although the ratio of apoC-III_1_ / apoC-III_0a_ was associated with greater TG concentrations (r = 0.35, p <0.001), the association of apo-CIII_1_ /apoC-III_0b_ ratio and TG concentrations was not significant (r = 0.06, p = 0.38). Total plasma apoC-III concentration was measured in a subset of samples at the upper (HC-III) and lower (LC-III) quartiles of the apoC-III_1_ / apoC-III_2_ ratio (n = 72). The individual concentrations of apoC-III proteoforms were then computed by multiplying the total concentration with the relative abundance of each protein. The concentrations of apoC-III_0b_ and apoC-III_1_ were highly correlated (r = 0.89, p<0.001), suggesting that the glycosylation of apoC-III (C-III_0b_) and glycosylation and sialylation of this protein (apoC-III_1_) are result of the same process, and explaining the lack of correlation of the ratio of apoC-III_1_ / apoC-III_0b_ with fasting TG. The concentrations of apoC-III_0a_ were also correlated with the concentrations of apoC-III_0b_ (r = 0.55, p<0.001) and apoC-III_1_ (r = 0.6, p<0.001). However, concentrations of apoC-III_2_ did not correlate with any of the other apoC-III proteoforms suggesting that a different mechanism regulates the addition of the second sialic acid to apoC-III.

**Fig 6:**
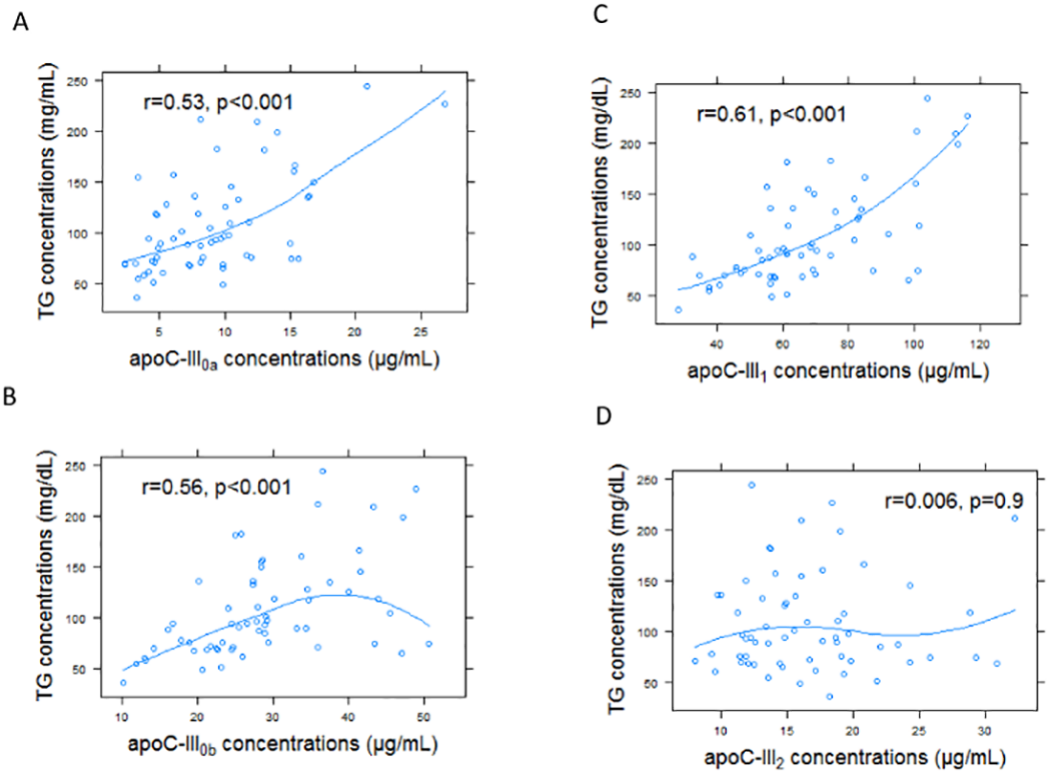
Relationship of apoC-III proteoforms concentrations and plasma TG concentrations (n = 72). This association was driven by apoC-III_0a_ (r = 0.53, p<0.001, A), apoC-III_0b_ (r = 0.56, p<0.001, B) and apoC-III_1_ (r = 0.61, p<0.001, C), but not apoC-III_2_ (r = 0.006, p = 0.9, D).

ApoC-III proteoform concentrations were examined in relation to fasting plasma TG. As expected, total apoC-III concentrations were strongly correlated with plasma TG concentrations (r = 0.63, p<0.001). The association of apoC-III proteoforms with TG was driven by apoC-III_0a_, apoC-III_0b_, and apoC-III_1_ but not apoC-III_2_ concentrations (Fig 6A, 6B, 6C and 6D). These data indicate that apoC-III_0a_, apoC-III_0b_ and apoC-III_1_ were better indicators of fasting TG levels than apoC-III_2_. Using a linear multivariate regression model with apoC-III concentrations and ratios as predictors of TG concentrations, an independent association between apoC-III concentrations and the ratio of the other proteoforms to apoC-III_2_ with TG concentrations was demonstrated (p<0.001 for all). For example, for one SD increase in total apoC-III concentrations, there was a 23 unit (standard error 5, p<0.001) increase in TG concentrations. For one SD increase in apoC-III_1_ / apoC-III_2_ ratio, there was 17 unit (standard error 4.8, p = 0.001) increase in TG concentrations.

## Discussion

ApoC-III in the plasma circulates predominantly as a sialylated apolipoprotein containing one or two molecules of sialic acid. ApoC-III is a key regulator of TG metabolism; however, the roles of the individual apoC-III proteoforms are not well understood. In the present study, we found that overweight and obese individuals had greater ratios of apoC-III_0_ or apoC-III_1_ proteoforms to apoC-III_2_ compared with leaner individuals. These ratios negatively correlated with insulin sensitivity (S_i_) and positively correlated with TG concentrations. Concentrations of apoC-III_0a_, apoC-III_0b_ and apoC-III_1_, but not apoC-III_2_, correlated with fasting TG. These findings indicate that apoC-III_0_ and apoC-III_1_ are under metabolic control with a potential role for decreased insulin sensitivity in their formation, independent of changes in obesity. Our findings indicate that apoC-III_2_’s effects on TG metabolism differ from the other apoC-III proteoforms.

The majority of apoC-III is sialylated at the Thr-74 residue through a process of O-linked glycosylation [18]. Sialylation is an intracellular process driven by sialyltransferases, a family of Golgi-membrane bound enzymes [36]. The exact sialyltransferases or sialidases that modulate apoC-III sialylation remain to be identified, but two candidate sialyltransferases are α2,3-sialyltransferase, that can add sialic acid to galactose in position 3’ (ST3Gal-I), and α2,6-sialyltransferase that can add sialic acid to GalNAc in position 6’ (ST6Gal-I). Human GWAS study implicated GALNT2 as an enzyme that regulates HDL and TG metabolism [37], potentially by modulating apoC-III glycosylation [38]. Carriers of GALNT2 gene variants have altered apoC-III sialylation patterns [28]. In addition, GALNT2 expression is under glycemic control [39]. Neuraminidase, a lysosomal enzyme responsible for removal of sialic acid residues of proteins, has been detected in the circulation. However, desialylation activity via this enzyme in plasma is unlikely, since the optimal pH for the reaction ranges from 4–5 [20]. Roghani et al [19] demonstrated that apoC-III sialylation is not essential for its secretion or packaging into VLDL using a cell line expressing Thr-to-Ala 74 apoC-III mutant. It is thus likely that apoC-III sialylation is a variable and nonobligatory step for further protein processing and secretion [20].

Our study is novel for the following reasons: (1) we used a high throughput mass spectrometry-based technique to identify apoC-III proteoforms in plasma; (2) we examined an obese group not taking any medications that can alter lipid physiology and without the confounding effects of type 2 diabetes hyperglycemia; (3) our obese study group was assessed for measures of insulin sensitivity (which is a factor that regulates apoC-III expression); and (4) the study highlights important changes in lipid metabolism with obesity that appear early in adolescence with potential implications for diabetes complications later on. Previous studies using different populations and techniques present conflicting results on the role of apoC-III sialylation in TG metabolism. In particular, lipid therapies (statins, fish oil, fibrates and niacin [26, 40]) and metformin [22] have been reported to increase apoC-III sialylations confounding the interpretation of these results. In agreement with our findings, Savinova et al [26] found relative decreases in apoC-III_2_ to apoC-III_1_ in patients with metabolic syndrome compared to controls. In addition, an earlier study by Falko et al demonstrated that high carbohydrate diet preferentially increases apoC-III_0_ but not the other proteoforms [24]. Dietary weight loss or gastric bypass reduced the ratio of apoC-III_1_ / apoC-III_2_ [21, 22]. In contrast to our findings and those of others [24, 26, 40], two other studies reported an association between increased apoC-III_2_ and type IV hypertriglyceridemia [17, 41]. This discrepancy could have resulted from examining different populations, the use of lipid altering therapies and by the use of different methodologies (such as isoelectric focusing) to assess apoC-III sialylations.

Although a minor fraction of apoC-III is carried by LDL, increases in apoC-III on LDL were observed in diabetes [42]. These changes are considered atherogenic [42], as apoC-III on LDL associates with small dense LDL formation [18]. We are currently investigating the hypothesis that more efficient liver uptake of sialylated apoC-III on VLDL enhances TG metabolism and limits its conversion to LDL, resulting in the appearance of apoC-III on LDL. This is a potential mechanism that can explain the relation between apoC-III_2_ and plasma TG.

Recruiting healthy controls in pediatric research is challenging, yet we were able to recruit a small number of normal weight participants. Our control subgroup, however, was small in size, with younger individuals, and fewer males than the overweight and obese subgroups. The differences in apoC-III proteoform patterns persisted after adjusting for age and sex. Our study was conducted in a younger Hispanic population and generalization to different age groups and ethnicities requires additional studies. MALDI TOF MS affects the carboxyl groups in glycosylated proteins and causes their loss, therefore it is considered less efficient in analyzing glycans that contain carboxyl groups (i.e. sialic acid contains one carboxyl group). The majority of studies which report this issue are focused on analyzing isolated glycans, or glycans from glycosylated proteins obtained after enzyme digestion, rather than looking at them in the complex structure of the protein [43]. In addition, literature confirms that loss of sialic acid in glycans is common when MS/MS analyses are performed [43] (which was not the case for our sample set). The benefit of MSIA (used in our study) is that the glycated proteoforms are analyzed intact, and in a very short time (to complete 5000 mass spectra average takes less than 15s). In addition, all samples throughout the analysis were treated in the same way (same laser intensity for ionization). A control plasma sample was analyzed in triplicate with each run and relative percent ratios for all apoC proteoforms (including the sialylated and asialylated apoC-III) were compared between the different runs. The variability between the signals from apoC-III proteoforms that contained zero, one and two sialic acids was <10%. Neither apoC-III_0a_ nor apoC-III_0b_ have sialic acid in their structure. The low abundance of the native (non-glycosylated apoC-III proteoforms—apoC-III_0a_) as opposed to the glycosylated apoC-III is in accordance to previous literature data [44]. Furthermore, Bondarenko et al [44] demonstrated similar profiles of the apoC-III sialic acid proteoforms with both MALDI and ESI-TOF (which is a softer ionization technique), indicating that the MALDI ionization did not induce removal of sialic acids in intact apoC-III..

We conclude that apoC-III proteoforms are associated with obesity and insulin signaling with apoC-III_2_ showing a different pattern of association. ApoC-III_2_, unlike the other apoC-III proteoforms, did not associate with greater fasting plasma TG. Our findings support kinetic studies to examine how the different proteoforms regulate VLDL clearance. Measuring plasma apoC-III sialylation ratio (such as the ratio of apoC-III_1_ / apoC-III_2_) can provide important insights into the biology of TG metabolism in conditions such as obesity and metabolic syndrome.

The minimal dataset is included in the supporting documents (S1 Table).

## Supporting Information

S1 Table The dataset for the association of apoC-III proteoforms with clinical and biochemical measurements. (XLSX)
